# Codesigning a Mental Health Discharge and Transitions of Care Intervention: A Modified Nominal Group Technique

**DOI:** 10.3389/fpsyt.2020.00328

**Published:** 2020-04-21

**Authors:** Natasha Tyler, Nicola Wright, Andrew Grundy, Kyriakos Gregoriou, Stephen Campbell, Justin Waring

**Affiliations:** ^1^NIHR Greater Manchester Patient Safety Translational Research Centre, University of Manchester, Manchester, United Kingdom; ^2^School of Health Sciences, University of Nottingham, Nottingham, United Kingdom; ^3^NHS Derbyshire Healthcare Foundation Trust, Derby, United Kingdom; ^4^NIHR School for Primary Care Research, Manchester Academic Health Science Centre, School for Health Sciences, University of Manchester, Manchester, United Kingdom; ^5^Health Services Management Centre, University of Birmingham, Birmingham, United Kingdom

**Keywords:** nominal group technique, mental health, discharge, care transitions, adverse outcomes, psychiatric discharge, intervention, information sharing

## Abstract

**Background:**

Discharge from acute mental health services has long been associated with mortality, risk, and related adverse outcomes for patients. Many of the interventions that currently aim to reduce adverse outcomes focus on a single group of healthcare professionals within a single healthcare setting. A recent systematic review highlights very few robust interventions that specifically aim to improve communication across services. However the importance of promoting interagency working and improving information flow between services is continually highlighted as a key priority.

**Methods:**

Using a novel codesign and experience based approach we worked with a multistakeholder group to develop possible solutions to reduce the adverse outcomes commonly associated with discharge from acute mental health services. This utilized a modified Nominal Group Technique and creative problem solving method to follow a four-stage process: Problem Identification, Solution Generation, Decision-Making, Prioritization and Implementation. Thirty-two healthcare professionals and an expert by lived experienced engaged with the process that took place over two stakeholder events.

**Results:**

Stakeholders at the first event identified and agreed upon 24 potential ideas to improve discharge from acute mental health services. These were refined at the second event to four elements of an interagency intervention: a multiagency ‘Discharge Team’ (with designated discharge coordinator), inclusive technology enabled team meetings, universal documentation and a patient generated discharge plan.

**Conclusion:**

This is the first study to codesign an interagency mental health discharge intervention based around a discharge team. We developed a model for working that places a greater focus on a patient generated discharge plan, interagency working, and information flow. A pilot of the proposed intervention is now needed to test the feasibility and effectiveness in reducing adverse outcomes.

## Introduction

Discharge from acute mental health services is often described as a dangerous, chaotic, and emotionally driven time-period for patients (1). This time period has long been associated with mortality, risk, and related adverse outcomes for patients (2–5). In England, between 2006 and 2016, there were 2,220 suicides within 3 months of discharge from inpatient mental health care (2). Discharged individuals are at much higher risk than the rest of the population for a range of fatal and nonfatal adverse outcomes (5). For example, a recent cohort study in Denmark found that within 10 years of their first discharge, 37.1% of males will have died, harmed themselves, committed a violent crime, or been hospitalized due to interpersonal violence. This population is also at increased risk for homelessness (6), is predicted to have a 15–20 year shorter life expectancy (7). In addition to more severe or long-term adverse outcomes, there are many less severe outcomes that can happen during the transitional period from acute services, such as loneliness, stigma, isolation, inability to access relevant community services, readmission within a short period, self-injury/harm, medication issues (8).

In the UK National Health System (NHS), many mental health trusts have adopted functional splits to inpatient and outpatient care, whereby different services lead care and treatment with an individual in different phases of the care pathway (9), which has arguably led to operational silos. Beyond this, patients are often discharged to different services depending on their individual needs; some may be discharged to an NHS service while others may need different social care services according to their needs. Many of these services operate different information sharing services, and discharge is sometimes delayed on the ward while waiting for communication or availability from the community services.

Researchers generally agree that if interagency communication and knowledge sharing were improved, adverse outcomes could be reduced (2, 5, 8, 9). The National Confidential Inquiry into suicide and self-harm recommends creating safer wards and early follow-up to reduce suicide following mental health inpatient admissions (2). Past research describes ‘improved interagency liaison’ as a means of reducing adverse fatal and nonfatal outcomes in this population (5). It has also been suggested that sound informational continuity between mental health and primary care or other providers has the potential to improve the efficiency, safety, and quality of care (10). Very little research considers the effects of improved or suboptimal interagency working/communication following discharge from acute services. However, research into other clinical populations has highlighted that safe hospital discharge relies upon effective communication and coordination across multiple organizations and boundaries (11). For example, research in operating departments shows that interdependencies and close working with other departments can improve safety (12). It is also recognized that there is little analysis of the threats to safety located within the wider system of care, especially between different care providers, processes, and settings (13). Past literature has described health services as a complex system involving a great number of dynamic, nonlinear interactions between multiple heterogeneous systems, actors, and units (14–15). Therefore, discovering means of improving interagency operations and communication at discharge could improve safety and reduce adverse events in this critical period.

Our recent systematic review highlighted 45 diverse interventions that have been developed internationally to address the problems associated with discharge from acute mental health settings (8). Some of these interventions focused on a particular threat to safety, such as medications management (16, 17). No intervention explicitly or exclusively focused on improving interagency working or communication (8). Numerous interventions instead encompassed a ‘boundary spanning’ element or agent, (*i.e.* a ward nurse in the community or a community nurse attending ward meetings) (18–20), which often had implications in terms of reducing readmissions (8, 21). When studying stroke populations, researchers found that no single transition intervention can facilitate knowledge transfer and that a combined approach is often needed focused on three main elements: 1) information systems/technologies (an effective system that allows for the transfer of explicit knowledge), 2) roles (dedicated professional roles to support discharge planning), and 3) group activities (interprofessional or interorganizational group activities) (22).

By facilitating conversations between the different agencies and individuals associated with inpatient to community discharge in mental health, it may be possible to (1) encourage interagency working, (2) identify mutual problems, and (3) provide a method to collectively develop mutually beneficial solutions. This paper describes how we used a modified Nominal Group Technique (23) to garner the collective knowledge of patients and various cadres of professionals who are involved in facilitating or supporting the care transition from acute mental health to community care using a creative, problem solving technique. A particular focus was given to identifying the interagency working problems and developing potential collaborative solutions.

From both a clinical practice and a theoretical perspective, to improve information sharing, it is important to understand how communication (exchange of information around a given task or activity) or knowledge sharing (the exchange, use, and assimilation of situated know-how) might ordinarily happen across inter-organizational and interprofessional boundaries (22). Work by Carlile proposes that knowledge boundaries between organizations and occupations can be understood in terms of their ‘difference’ and ‘dependencies’ (24). Difference relates to the different forms of knowledge that groups hold and also the knowledge they need (*i.e.* the extent of expertise around a given problem); whereas dependency relates to the extent that the knowledge of another group is needed. Carlile theorizes that when differences between groups are small and dependencies known and agreed upon, transfer of knowledge can be standardized through ‘syntactic knowledge transfer’, for example, developing a common language or common information system (24). However, when differences are more significant there is a need for ‘semantic knowledge transfer’, whereby meanings, beliefs, and assumptions must be translated across the knowledge boundary. To further understand the differences and dependencies in this diverse professional group, it is essential to bring multidisciplinary and interorganizational professionals together and create a space to explicitly discuss knowledge differences and dependencies (*i.e.* what knowledge each group has and what they need and whether any potential interventions need to involve any translation of knowledge across boundaries, *i.e.* somebody to translate into a common ‘language’).

The study aimed to produce an intervention to improve transitions of care by eliciting a core set of priority issues. The main research question related to whether it was possible to obtain consensus from the views of various stakeholders across mental health and social care provision within a single NHS trust and the organizations associated with it and to use the rankings of highest priority to create the basis for an intervention to improve communication and interagency working.

## Methods

### Study Design

The Nominal Group Technique (NGT) was originally developed as an organizational planning tool in 1975 (23). It has since been used in many fields and widely in healthcare settings, for example, evaluating education or enabling problem identification or solving (25, 26). NGT involves both quantitative and qualitative data collection generally in small groups of stakeholders and traditionally involves four steps: (i) silent generation of ideas individually; (ii) round robin recording of ideas; (iii) structured and time limited discussion of the ideas; (iv) selection and ranking of ideas (27). There are numerous interpretations of the steps, and the initial model proposed by Delbecq traditionally had three broad distinct stages: 1) problem identification, 2) solution generation, and 3) decision making (28). To our knowledge a Nominal Group Technique has not been used previously to bring together stakeholders from acute and community mental health services to collectively identify problems and coproduce solutions and feasible interventions.

We used a modified Nominal Group Technique with an explicit creative problem solving technique (29). The Nottingham Ingenuity Process is based on the principles of Nominal Group Technique but includes a number of modified elements. The *Nottingham Ingenuity Process* is based on the principles of entrepreneurship and collective intelligence as a means of solving problems that businesses or individuals face (29). The modified elements that differentiate our chosen method from traditional Nominal Group Technique are: (i) the introduction of a ‘critical friend’ in phase 3; (ii) the decision to present three ideas related to implementation time-markers at the end of the process; and (iii) a decision to split the process across two days with a reduced participant group in the latter. A potential solution to problems surrounding discharge from acute mental health settings was codesigned with multidisciplinary participants from primary/secondary care, emergency services, community/social services and a lived experience expert. Data were collected on two separate days six weeks apart. Problem identification and solution generation happened at event 1, and event 2 focused on decision making and implementation.

### Setting

The event was held within a single United Kingdom (UK) National Health Service (NHS) Trust and associated services. The NHS Trust is a Mental Health based services, however, does support the practice of children’s services and health visitors. Currently the patient population the Trust covers is 800,000. The Trust supplies care for people with mental health needs of a variety of degree including, inpatient acute mental health of a working age, older adults of both functional and organic nature, forensic inpatient and community services, rehab services, community mental health services, substance misuse services, and psychological services. The NHS Trust at present has eight inpatient acute wards for people of a working age. Each ward ranges from 20 to 22 patients and range between mixed sex or single gender wards. Due to the nature of the acute inpatient wards there is significant contact with external services including other emergency services such as ambulance and police and with other social based services including Approved Mental Health Practitioners. Within mental health services there is a high proportion of patients who come across challenges with their employment and accommodation, and as a result, the ward teams engage a large amount with social care services, enablement teams, accommodation services, and homeless services.

#### Event 1

The full-day event was hosted by a facilitator from the *Haydn Green Institute* at the University of Nottingham, who specialized in the *Nottingham Ingenuity Process*. **Table 1** shows how this relates to traditional NGT methods. The event began with an introduction to the project and the work so far, outlining interventions from the systematic review (8) and a presentation about the patient perspective. Participants were split into five round-table groups, evenly distributing ward and nonward professionals.

**Table 1:** A table to highlight the relationship between the stages of the Nottingham Ingenuity Process and Traditional Nominal Group Technique.

Nottingham Ingenuity Process Stage	In line with Delbecq Nominal Group Technique Stage (23)	Activities
1. Define	Problem Identification	Outlining the problems
2. Discover	Solution Generation	Silent generation of ideas
3. Determine	Decision Making	Recording of ideas, discussion, decision making, prioritizing
Modified Stage (not part of Nottingham Ingenuity Process)
4. Prioritization, Implementation and Intervention Development	Decision Making	Further reducing and prioritizing ideas generated in phase 3.

##### Phase 1: Problem Identification

In the initial phase we asked participants to work in small groups of three to six participants to outline the problems associated with communication and interagency working relating to discharge from acute mental health to community services.

##### Phase 2: Solution Generation

In phase two we asked individuals to silently generate as many potential ideas as possible, without being bound to notions of feasibility, finance, or execution. Ideas were recorded on ‘post-it’ notes and attached to a flip-board for discussion in the next phase.

##### Phase 3: Decision Making

The decision making phase had multiple components: 1) recording of ideas collectively; 2) discussion of the individual ideas generated; 3) deciding what is feasible using three time markers a) today, b) next month, c) in 6 months; 4) moving two people from each group to play the role of a critical friend to discuss feasibility of potential ideas; 5) deciding which of the potential ideas are the three to five priorities they would like to present to the wider group. One participant from each group verbally presented their ideas to the wider group, and the presentations were audio-recorded, with written consent obtained.

#### Event 2

##### Phase 4: Prioritization, Implementation, and Intervention Development

The half-day prioritization, implementation, and intervention development event began with a presentation of the ideas from event 1 and where relevant, how ideas related to academic literature. The titles of all potential ideas were also displayed around the room. There were then four distinct elements: (1) idea reduction: reducing the long list of items from the last event to a manageable, feasible list of 10 items through small group discussion; (2) individual ranking in silence of the top five preferences from the list; (3) discussion of group rankings and generation of best three ideas (including a visualization exercise using sticky dots to triangulate voting findings); and (4) discussion of how the organization might implement these three interventions in practice. We chose to rank the top five preferences as it is in line with other literature and guidelines, although there is no predefined number (23, 30).

### Participants

#### Event 1

We used a convenience sample, initiating with a single UK NHS trust comprising of two campuses; we then snowballed sampled individuals who interacted with the acute mental health wards of this trust. We aimed for 50% of the participants to be staff members (of any cadre) from acute mental health settings and 50% as staff members from community, primary or social care, emergency services or had lived experience expertise. As the intervention aimed to focus on communication between the acute ward and other services, we aimed to ensure there was adequate representation from acute ward staff in each of the small group discussions. The research team developed a protocol (see **Supplementary File 1**) outlining preferable numbers of each professional cadre and organization to generate a diverse stakeholder group. When a participant dropped out of one group we aimed to find a participant of similar professional background. Participants received a £20 gift voucher and travel expenses in recognition of their time.

#### Event 2

At event 2, we chose to invite a smaller multiagency, multiprofessional sample to enable meaningful discussion (n = 10). We invited a selection of key professionals including lived experience expert, police officer, social worker, nurses, consultant psychiatrist, and operational acute setting management and primary care healthcare professionals. There were 15 professional groups in attendance at event 1, and we chose the smaller to ensure a mixture of health and social care professionals and a mixture of ward and community organizational staff, based on practical considerations of who was financially and feasibly able to attend (see **Supplementary File 1**).

### Data Sources

Relevant data from phases one, two, and four were collected from participant’s written notes by each group and compiled into tables. Audio-recorded presentation of findings by each of the small group representatives were collected in round three; the recording was later transcribed then tabulated to highlight the 24 distinct ideas. In phase four, anonymous rankings were collected and calculated independently by two researchers (NT, JW). Hand-written notes were taken about prioritization and implementation discussions by two researchers (NT, NW) and were used solely to provide context to any ranking/voting results. The qualitative data collected was not analyzed further as the purpose was solely to enable accurate recording of ideas/rankings as opposed to in-depth qualitative analysis, which happened at other points within this project which were designed and sampled purposefully.

## Results

### Participants

#### Event 1

Thirty-two participants attended event one. As anticipated, the mixed professional and organizational group was almost equally split between ward and community/social/primary care staff. Fourteen participants were based primarily on acute wards, including ward administrators, entry level nurses, senior management, and consultants. Seventeen professionals worked in a primary, community, or social care role, including emergency services, local authority housing services, general practitioners, and community nurses. One participant was an expert by lived experience. Across the organizations, professionally 13 were in a nursing role, seven in a service management role, two doctors, two police officers, two social workers, two ambulance staff, two administrators, and one occupational therapist. **Table 2** shows the role and organization of each participant. Six participants were due to attend on the day and did not, two worked for specialist mental health accommodation services (manager and administrator), four were trust staff healthcare professionals based on acute wards (nurse, two healthcare assistants, occupational therapist).

**Table 2:** Participant Demographics (Organization, Role).

Participant Number	Organization/Location of work	Job Title
1	Crisis Team	Service Manager
2	Acute Ward	Ward Manager
3	Acute Ward	Ward Occupational Therapist
4	Crisis Team	Clinical Lead Nurse
5	Liaison Team	Nurse Consultant
6	Acute Ward	Consultant Psychiatrist
7	Acute Ward	Lead Nurse
8	Acute Ward	Lead Nurse
9	Acute Ward	Nurse
10	Crisis Team and Acute Wards	Housing Worker
11	Acute Ward	Ward Administrator
12	Across Trust	Head of Nursing
13	Across Trust	Service Manager
14	Across Trust	Out of Area Case Manger
15	Criminal Justice and Liaison	Acting Service Manager
16	Across Trust	Assistant Head of Nursing
17	Ambulance Service	Clinical Navigator
18	Police Service	Mental Health Co-Ordinator
19	Ambulance Service	Clinical Navigator
20	Primary Care	Specialist MH Nurse
21	Primary Care	Specialist MH Nurse
22	Primary Care	General Practitioner
23	Community Care	Community Psychiatric Nurse
24	Council Services	Manager Homelessness
25	Social Services	Social Worker
26	Crisis Team	Social Worker
27	Police/Community Service	Police Officer
28	Crisis Team	Senior Nurse
29	Crisis Team	Lead Nurse
30	Police/Community service	Lead Nurse
31	Rehab and Community service	Lead Nurse Rehab and Recovery
32	University of Nottingham	Lived Experience Expert

#### Event 2

Eight selectively sampled individuals attended event 2. Participants were of mixed professional backgrounds and were located both within the NHS trust and external organizations. Two professionals could not attend on the day, one lead nurse from an acute ward and one primary care mental health nurse. **Table 3** shows the roles and demographics of the participants who attended event 2.

**Table 3:** Role and organization information about participants at event 2.

PP Number	Role	Organization
1	Head of nursing	NHS Trust
2	Nurse	NHS Trust
3	Nurse	NHS Trust (Crisis Team)
4	Consultant Psychiatrist	NHS Trust
5	Operational Manager	NHS Trust
6	Lived experience expert	University of Nottingham
7	Social Worker	Social Services
8	Police Mental Health Engagement Officer	Police

##### Event 1, Phase 1: Problem Identification

Between the five groups, 47 unique problems were identified, the majority of which were distinctive to each individual group. Nine problems were identified between two or more groups. **Table 4** shows the most commonly identified problem was lack of resources (human and financial) enable greater interagency working and ‘fear’ about repercussions if a wrong decision is made about discharge (*i.e.* risk aversion) was the second. The items most common and relevant to our research topic of interagency working and communication were: lack of clarity about expectations of each group, silo working, no multiagency processes and no-one taking responsibility for coordinating the transition.

**Table 4:** Problems identified by stakeholder groups and the numbers of group that identified each.

Problem		Number of groups that identified
1	Lack of resources (human/organizational finance to enable better inter-agency working)	**4**
2	Fear (including risk aversion, fear of repercussions for wrong decisions made about discharge and adverse outcomes)	**3**
3	Lack of clarity about expectations of each group (including patients and carers)	**2**
4	Blame culture (including fear of blame)	**2**
5	SILO working (lack of information sharing)	**2**
6	Ineffective communication and interagency working	**2**
7	No multiagency/disciplinary processes	**2**
8	No one taking responsibility for coordinating the transition (Lack of admission/discharge co-ordinator)	**2**
9	Not planning discharge from admission	**2**
10	Target driven culture	**1**
11	Excess paperwork	**1**
12	Ticking boxes	**1**
13	Law of unintended consequences (not learning from it and reviewing and adapting)	**1**
14	Missing opportunities to create therapeutic environment	**1**
15	Expectations of regulatory bodies	**1**
16	No flexibility within referral pathways	**1**
17	Micro-managing staff	**1**
18	Patient blaming (positive risk management)	**1**
19	Internal/external/partner agency communication	**1**
20	Duplication	**1**
21	Lack of involvement of patient, family/carers in information sharing	**1**
22	Patient/carer/other not being communicated what/who/why	**1**
23	Lack of multiagency/disciplinary strategy	**1**
24	Lack of multiagency/disciplinary meetings	**1**
25	Few interagency links	**1**
26	Lack of right care at the right time in the right time	**1**
27	Inappropriate admissions	**1**
28	Lack of educational/community resources	**1**
29	Insufficient beds	**1**
30	Pressure to discharge	**1**
31	Inappropriate cluster 7 and 8 provision	**1**
32	Austerity	**1**
33	Hierarchical Healthcare (top-down structure)	**1**
34	Defensive practice	**1**
35	Choice of wording—discharge	**1**
36	Information from MHA not being relayed to hospital, social worker *etc*.	**1**
37	No list of agencies that need to be contacted	**1**
38	Agencies working on different systems	**1**
39	Information not being relayed to ward staff at admission (from community agencies)	**1**
40	Revolving door	**1**
41	A and E breach reporting	**1**
42	Tension between teams (crisis, inpatient, CMHT)	**1**
43	Lack of alternative to inpatient admission	**1**
44	Insufficient early discharge planning meetings	**1**
45	Insufficient care planning in community (advanced statements *etc*.)	**1**
46	No ethics committees or complex case panels	**1**
47	Care pathways unclear	**1**

##### Event 1, Phase 2: Solution Generation

There were 395 potential initial ideas generated across the groups. These range from high organizational level solutions, such as a complete change of service provision, (*i.e.* a mental health walk in center), to infrastructural (*i.e.* a shared information system), to cultural (*i.e.* starting discharge planning from admission).

##### Event 1, Phase 3: Decision Making

After a small group discussion and reduction of ideas, 24 unique ideas were presented to the whole group. These were considered feasible to deliver and implement either the next day, within the next month, or within six months. Ideas included large-scale ideas, such as the introduction of recovery colleges, to smaller scale interventions such as multiagency meetings. These 24 ideas were taken forward to event two for further discussion and prioritization. **Table 5** presents the distinct solutions developed by the five small, multiprofessional teams.

**Table 5:** The 24 ideas generated after event 1.

Idea 1: Little Red Book (refers to personal child health record, patient held records, used to carry information between services when a child is born in the English and Welsh National Health Service)
Idea 2: Crisis and Respite Admissions (enabling individuals to have admissions to acute wards for respite periodically without usual referral processes)
Idea 3: Nurse-led Discharges (criteria-led discharge, enabling nurses to discharge patients to reduce delays to discharge awaiting consultant decisions)
Idea 4: Discharge Teams (multiorganizational teams that meet periodically to discuss transitions of care)
Idea 5: Patient Writes Discharge Plan (a discharge plan led by the needs of the patient, this may be in additional to clinical plans)
Idea 6: Mental Health Coordinator in each GP practice (a professional responsible for co-ordinating care and signposting for individuals with mental health problems, not necessarily a clinician)
Idea 7: Building Professional Relationships (a program of activities that focuses on building direct professional relationships between staff in different organizations)
Idea 8: Starting Discharge Planning from Admission (an initiative that encourages ward staff to plan for discharge when the patient is admitted)
Idea 9: MultiAgency Risk Management Plan (a risk management plan that can be used across agencies to reduce duplication of paperwork and also improve information flow between agencies)
Idea 10: Risk sharing between housing and hospital services (an initiative that encourages professionals in health and social settings to take joint responsibility for risk management, through joint procedures, information sharing/documentation, increased communication)
Idea 11: Multiagency Meetings (periodic meetings: face-to-face or technology enabled, between staff from all involved agencies to discuss patient transitions)
Idea 12: Patient Contracts (a coproduced contract that outlines expected behaviour from patients and staff)
Idea 13: Management Practice Weeks (a week where managers from each agency shadow their counterpart in another agency to understand their pressures and encourage relationship building)
Idea 14: Personality Disorder or Cluster 7 and 8 Pathway (a care pathway specifically for individuals with personality disorders and similar diagnosis)
Idea 15: Stepdown Service from Community Mental Health (a service between with care levels in between acute and community that enables higher levels of support and care than community to reduce feelings of loneliness/isolation post-discharge)
Idea 16: Purposeful Admission (ensuring there is a purpose for all admissions onto a ward, e.g. medications resolution)
Idea 17: Admission Avoidance Care Plan (a care plan that focuses on avoiding unnecessary admissions, by signposting other services and identifying triggers and ways of over-coming them in the community)
Idea 18: Zero Tolerance Redefinition (Zero tolerance is an English and Welsh National Health Service Policy to tackle violence against healthcare professionals)
Idea 19: Redefining MDT Meetings (a redefinition what a multidisciplinary team meeting is, who can attend, how often they should be, invitations of community, primary, social and emergency professionals where necessary)
Idea 20: Community Services Discharge Coordinator (a coordinator that is primarily based in the community and coordinates discharges, but that visits patients on the ward bridging the boundaries between community and acute care)
Idea 21: Personal Life Coach (introduction of a life coach service post-discharge that enables individuals to overcome psychosocial challenges associated with transitions from acute services)
Idea 22: Recovery College (an existing initiative that offers educational course for patients in mental health, to be offered post-discharge)
Idea 23: Self-referral to the Crisis Team (to enable patients to refer themselves to the crisis team, rather than through a professional agency)
Idea 24: Better understanding of other agencies through buddying and shadowing

##### Event 2, Phase 4: Prioritization, Implementation, and Intervention Development

The 24 ideas were deliberately reduced to the 10 most effective and feasible during group discussions. At this stage the group noticed that some ideas were very similar and it was decided that some would be combined, which left nine unique ideas to vote upon. The group voted unanimously to remove the 13 least effective or feasible ideas, for example removing those with past evidence of ineffectiveness or those not specifically focused on communication., **Supplementary File 2** shows the reasons for exclusions and inclusions, primarily around a) scope what is achievable/feasible to implement within the team of professionals at the event and b) specificity to discharge, hence introductions of new services or mental health initiatives that did not focus on discharge were excluded.

Of the nine ideas, the three highest ranking ideas were: 1) multidisciplinary discharge teams with designated discharge coordinators in each relevant agency; 2) patient writes own discharge plan; 3) multiagency risk management. **Table 6** shows the intervention ideas, the individual scores allocated to each, and the total score.

**Table 6:** The proposed intervention ideas, the scores provided by each participant and the total score.

Intervention ideas	Cumulative ranks
4. and 20. MDT Discharge Teams with discharge coordinators on the acute ward and representatives from each of the community services	26
5. Patient Writes Discharge Plan	25
9. Multi agency Risk Management	20
11 and 19. More inclusive multiagency meetings with technology	12
2. Crisis and Respite Admission	11
24. Better Understanding of other agencies through buddying and shadowing	10
3. Nurse led Discharge	8
10. Risk Sharing between hospital and housing services	6
7. Building better relationships between agencies	2

Each participant had the opportunity to rank their most favored five ideas. The idea they most agreed with was scored five, the fifth preference received a score of 1 and the remaining five ideas 0. Scores were calculated collectively to give a total score for each item across the group. The intervention numbers relate to the 24 ideas from event 1.

### Proposed Model of Working

**Figure 1:**
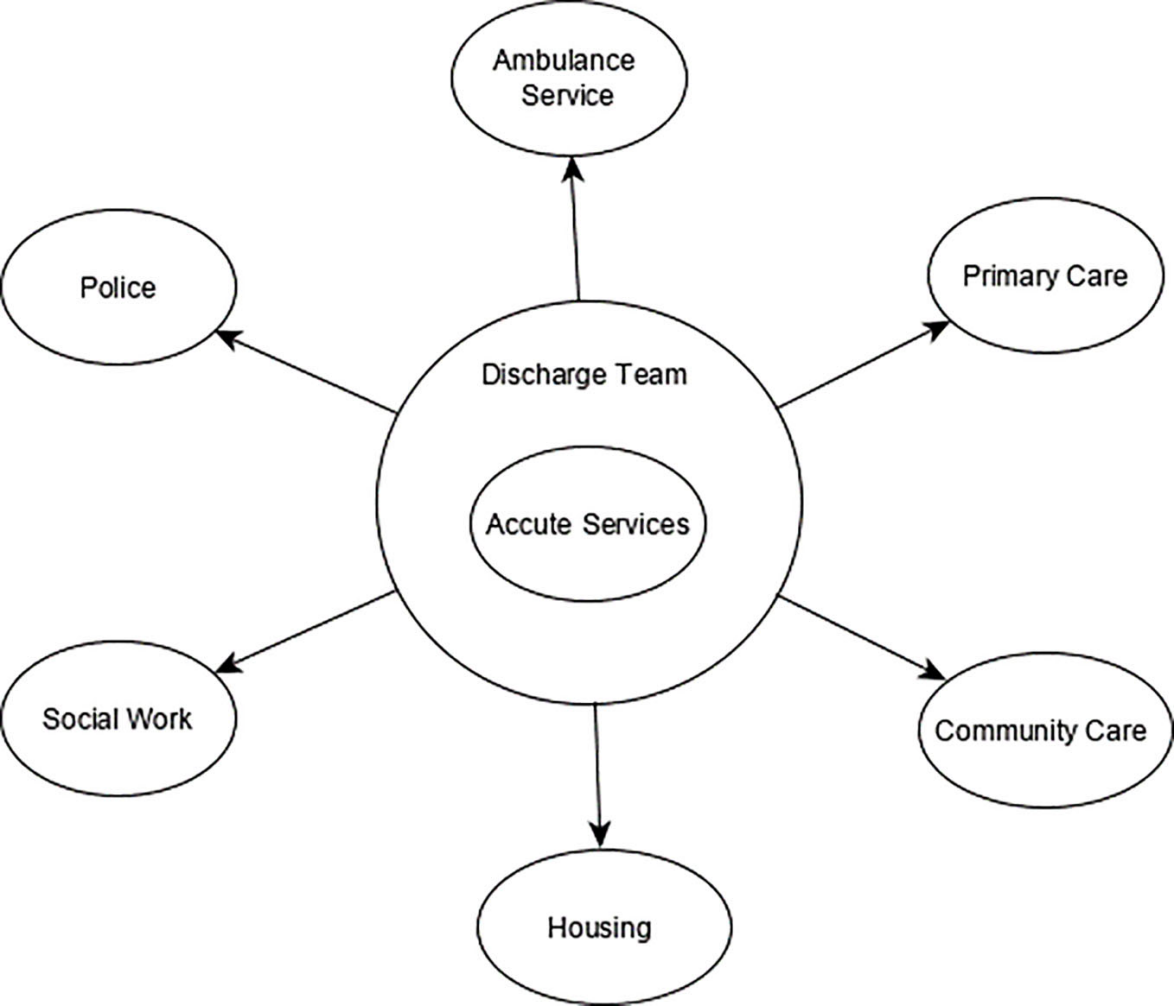
An example composition of the ‘discharge team’ intervention where each oval signifies a representative from each organization to attend frequent group meetings to discuss transitions of care. The group would be organized and led by a representative from the acute ward.

As a result of the modified Nominal Group Technique we devised a potential model of working (that can be taken forward and piloted as an intervention) that embodies the most highly ranked idea ‘Multiagency discharge teams with key agents within each organization’. As part of this intervention we incorporate elements of other highly ranked ideas to maximize the potential for improved communication and interagency working. **Figure 1** outlines a proposed example composition of the discharge team led by an acute discharge coordinator, within each locality. The team would communicate frequently remotely and meet face-to-face at regular predefined intervals (weekly, fortnightly or monthly to be decided in initiation phases). There would be a key contact within each organization, thereby improving direct communication and interprofessional relationship building. In implementation discussion professionals suggested the team would include a key discharge coordinator who is based on the acute ward and a clinician, but there may also be an administrative key contact as part of the team. As the key discharge coordinator would be in the acute ward, the main focus of this team would be transitions to and from the acute ward; however discussions around any other transitions would be encouraged so that all professionals have an idea about the position of patients within the care pathway.

**Figure 2:**
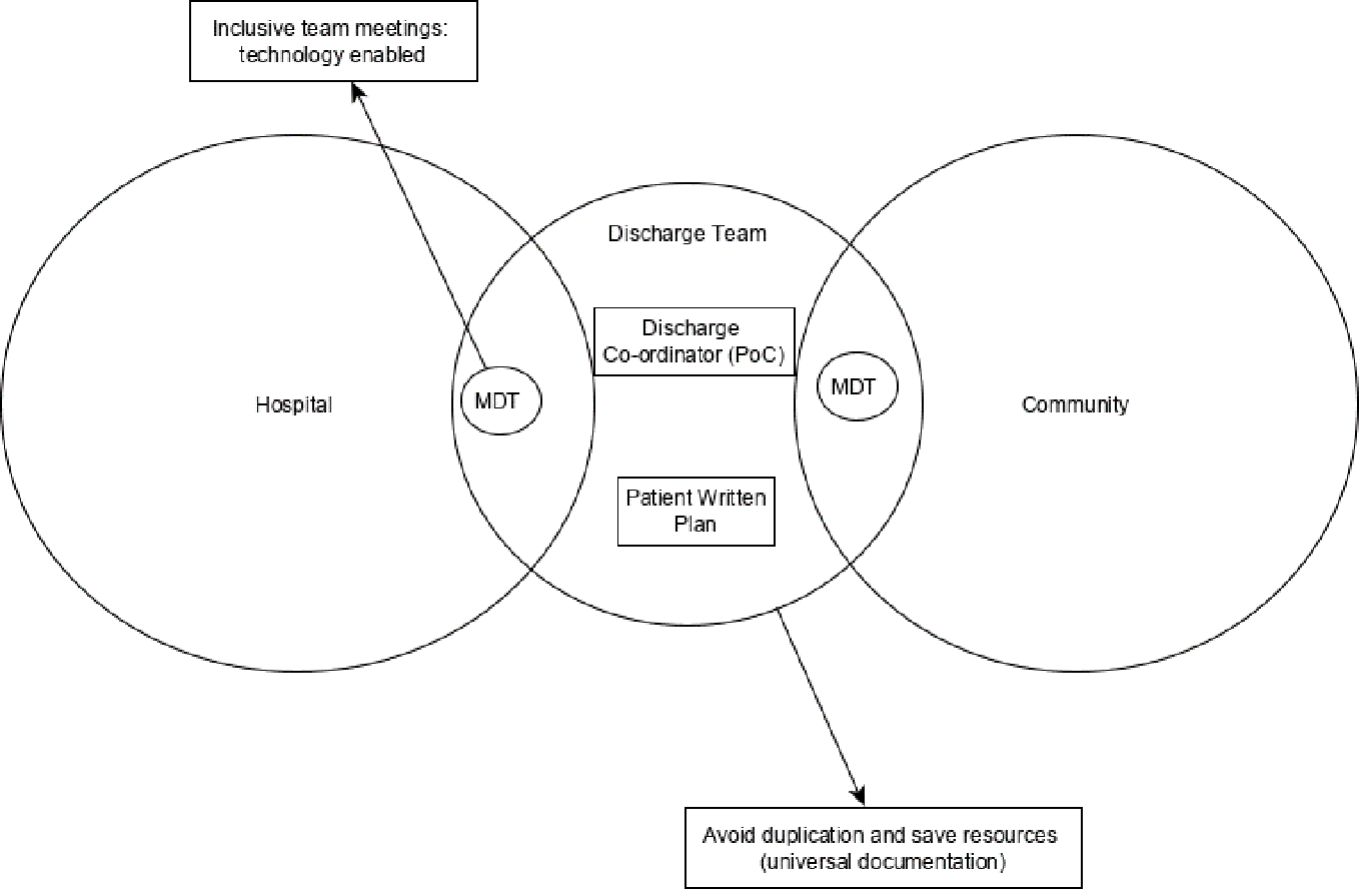
A diagram to highlight how the proposed interorganizational intervention might be composed. The outer circles represent the two care settings/environments and the discharge team (supported by other elements of knowledge sharing) would aim to reduce the epistemological, physical, and semantic boundaries between the two.

We also propose that the intervention would encapsulate elements of the other highly ranked ideas and solutions to initial problems: a) incorporating technology into multidisciplinary meetings (**i.e**. skype, conference calls), b) patient writes discharge plan, c) universal documentation (see **Figure 2**). Implementation discussions suggested that increased contact between agencies could be facilitated by planning inclusive meetings, but removing the barriers of physical location, time, and resources using technology such as video/conference calling between the members of the discharge team. The ‘patient written discharge plan’ was discussed as an intervention to improve patient knowledge and understanding of the discharge process and to also highlight to healthcare professionals what is important to each individual. However, there were discussions around the formatting of this intervention, and it was suggested that a blank page may be intimidating, as might too many structured questions, therefore it was agreed that the ‘tool’ used to format this discharge plan should be coproduced with patients. It was felt that a patient written discharge plan would improve knowledge sharing between healthcare professionals and patients by enabling patients to highlight the knowledge they require and communicate this across settings. Finally, while the focus of the group is primarily to operate at a level of individual patient care, the group would also provide a vehicle to facilitate system level improvement such as universal documentation. Universal documentation was an issue that was discussed in relation to improving multiagency risk management. Stakeholders felt that different organizations and professional groups had similar documentation that was repeated; they highlighted that the discharge team could work together to streamline this to reduce staff workload and patient fatigue. This intervention proposes a move towards making documents and systems universal across agencies, as a means of improving risk management and also reducing duplication.

From a feasibility perspective this work highlighted multiple implementation challenges; however these were expected to exist at a local level within each organization. Technology was seen as a key enabler to remove logistical barriers of travel and time away from duty. However, there were some discussions around barriers mainly due to resistance from organizations due to overworked staff; for example one patient suggested that GP involvement might prove problematic. It was decided that implementation would need to be discussed at a local level within the initiation stages.

## Discussion

We found 47 distinct problems identified by the diverse stakeholder groups. Shared challenges articulated by more than one group included lack of clarity about expectations of each professional group or agency, silo working, no multiagency processes, and no individual/team taking responsibility for coordinating the transition from hospital to community. To overcome these challenges the groups decided on 24 distinct potential interventions. In the second event, the groups decided that the most feasible and effective intervention (from the 24) would be the introduction of a multiagency discharge team with a key representative from each agency and a coordinator from the acute ward. This group would meet frequently to discuss care transitions, and technology would reduce barriers with travel and time commitment. Ideally, the discharge team would work towards some elements of universal documentation and patient written discharge plans (as these were highly ranked complementary interventions).

As there was little evidence of robust interventions that specifically aim to improve interagency working and communication between services (24), we chose to go directly to the multiagency professional group to attempt to elicit novel, intuitive or experience-based solutions to the problems associated with mental health acute service discharge. As a result of the modified Nominal Group Technique we have elicited many varied intervention ideas that could address some of the problems associated with discharge from acute mental health services. Furthermore, we were also able to ask healthcare professionals, other professionals and NHS service managers to articulate the ones they felt could be most feasible and effective and have articulated these as a combined potential intervention.

Much of the literature in our recent systematic review (8) problematized readmission, post-discharge suicide, medication management, and symptom management associated with discharge from acute mental health services (8, 17, 31). Yet we found that staff considered fear, blame, and clarity about each group’s expectations to be root-problems from an interagency perspective. Very little previous research into acute mental health discharge has looked at the underlying social and bureaucratic underpinning elements (fear/blame/expectancies) that could affect interorganizational working. This is likely to elicit a knock-on effect on patient safety in this vulnerable period of the care pathway. For example, a great body of literature exists highlighting the balance between blame and accountability and the effect this has on patient safety (32, 33), with the premise that most errors are committed by good, hardworking people trying to do the right thing (32). Engaging with these professional levels, underlying social and bureaucratic issues may have the potential to develop more meaningful interventions that increase quality and safety.

This NGT method highlights the social, cultural, and political undertones that are present, which must be considered when assessing successful integration of interventions into practice. The most feasible and effective solutions that were voted for in the final round mirror the findings in other clinical populations. For example, in hip and stroke patients, an ethnographic study found three successful care transition interventions: information systems, specific discharge roles, and group based activities and concluded that care systems should look to develop multiple complementary methods to improve interprofessional communication by encouraging dynamic knowledge sharing and learning. The ideas organically generated by professionals in our events predominantly mirrors the solution categories proposed in existing literature (information systems, discharge roles, and group activities).

The intervention that was developed as a result of the modified Nominal Group Technique is similar and potentially complementary to some of the interventions currently proposed in the literature. For example, past research has highlighted the importance of ward staff developing therapeutic relationships with patients that continue after discharge until a therapeutic relationship is established with community mental health professionals (20, 34, 35). Effective model that focuses on relationships between healthcare professionals and patients could be further reinforced by simultaneously strengthening multiagency professional relationships and communication between agencies through the introduction of a multiagency discharge team, strengthened information systems, and inclusive, technology enabled meetings. Similarly, some interventions in the literature propose the use of technology enabled meetings or communication, with varying success (8, 36, 37); however these may also be strengthened by establishing distinct discharge teams and involving professionals from many agencies and the patient.

Using a multiagency stakeholder team to support integration in care transitions has recently been successfully trialled in local initiatives in other clinical systems and populations. For example, a team in Cardiff reported great success in reducing frequent emergency department attendance by holding a monthly multidisciplinary, multiagency stakeholder group (including housing, police, social services, charities) (38). Given the success of multiagency meetings in other stakeholder groups to support integration, there is a potential that the mental health discharge team could also be successful. However, whether this intervention has an effect on reducing post-discharge outcomes that are more common in mental health populations (suicide, mortality, self-harm) would need to be tested.

Psychiatric discharge coordinators (single discipline/organization) have been trialled with limited success; for example one study found little improvement in outcomes with the introduction of a psychiatric discharge coordinator (39). As our intervention proposed a group-based approach rather than a model reliant on a single individual, there is potential that group-based discharge activities may provide a basis for more direct knowledge translation that may elicit interprofessional ‘bonding’ at a cultural and organizational level, as suggested in previous research, ultimately providing a more successful discharge intervention.

In terms of the effectiveness of the Nominal Group Technique as a method of developing mental health discharge solutions, we found that NGT can be an effective method of problem identification and solution generation in the interorganizational space of hospital discharge. The solutions generated were in line with much of the theoretical work conducted into effective discharge solutions (12), despite providing no theoretical guidance to participants. We particularly found this to be a useful method of addressing local problems, and participants reported positively about being able to engage with staff from other agencies and the networking and relationship-building opportunities it provided.

From a theoretical perspective, the Nominal Group Technique provided healthcare professionals with an opportunity to discuss differences and dependencies of knowledge and what is needed to bridge the knowledge boundaries between organizations and occupations (24). Carlile argues that when differences and dependencies are shaped by divergent political interests that impede knowledge exchange then it is important to promote more pragmatic knowledge exchange by creating a common agenda to address shared problems (24). Informally, event participants fed back that the opportunity to discuss shared problems with interorganizational colleagues (even as part of the NGT exercise) was beneficial. Therefore, using the NGT technique in other local initiatives may build the foundations for a common agenda and shared problem solving [which is considered instrumental by Carlile (24)] for care transitions bound by social, political, and economic interests. The results suggest that groups were able to generate their own shared problems with the NGT technique and also look at ways to solve them.

The NGT technique not only fostered group ownership of ideas generated but also collectively generated expertise and developed solutions that reduce the effects of knowledge differences and allow for smoother dependencies; for example the discharge team would generate new shared knowledge and remove the necessity to ascertain meaning or tacit knowledge from forms or information systems by facilitating face-to-face or technology assisted verbal communication. Without being explicitly made aware of knowledge sharing theory, the groups organically developed a solution that incorporates the three elements of effective knowledge transfer in care transitions highlighted in similar transitions literature (22). Participants proposed a discharge team, which encompasses the ‘group’ element of knowledge transfer, a primary discharge coordinator on the acute ward and an elected person in other organizations (this is in line with the role component). Finally, they proposed three information sharing/technology elements to the interventions: a patient written discharge plan, universal information systems, and technology enhanced meetings. This research strengthens existing argument that single solutions are not effective (22) by highlighting the importance of a multicomponent solution to information sharing in care transitions.

## Strengths and Limitations

This research focused on gathering the collective tacit knowledge of individual professionals working across multiple agencies in primary, secondary, and social care within a single English National Health Service Trust and associated organizations. A strength of this work is that it brought together the opinions of stakeholders across the transition process to codesign a solution, thereby reducing ‘silo’ working. However, as such, the result may lack generalizability to other trusts or other national and international systems. This solution focuses on identifying problems and coproducing solutions, but it is well-documented that social, cultural, professional, or political problems can vary considerably between organizations (40). While the study could be criticized as not all of the participants from event 1 were present at event 2 (8), both groups generally represented the constituencies of stakeholders that we were seeking to represent. However, at event 2 primary care health representatives were unable to attend, which could have affected the intervention decision particularly due to the greater number of primary care services/professionals that operate with a trust locality.

There is a limitation of only having one lived experience representative, and we acknowledge the important contribution that could have been made if more lived experience representatives and their families/carers had attended the event. There is a potential that one service user representative may have been overwhelmed by professional voices.

The proposed solution is somewhat simplistic, and there are a number of pragmatic issues that may thwart implementation, such as over-burdened staff not prioritizing group meetings. Therefore, it is recommended that if trialled, the intervention should be accompanied by a behavior change intervention (41) or normalization process model (42) to enable an interagency intervention to become part of normal practice across settings.

## Future Directions

Future research should consider whether transitional, multiagency interventions developed using NGT at a local level can be used to address problems on a national or international level. A pilot is now needed of the model as an intervention to test the effect that improved knowledge sharing may have on adverse outcomes associated with this population. Particular attention should be paid to the outcomes used to measure the effectiveness of a communication intervention, as appropriate selection of outcomes in mental health research is a particularly difficult task highlighted in current literature (43).

From a clinical and service provision perspective, individual trusts and associated services may be interested in applying the results of this research to practice by developing a ‘discharge team’ with key contacts from each organization to attend frequent meetings (*e.g.* monthly). Organizations may also want to allocate a clinician and/or administrator from acute services to facilitate and lead this team. They should try to explore the potential to facilitate the meetings with the use of technology (video/telephone attendance) as this was described as a barrier to implementation by professionals across the services.

## Conclusion

We worked with an interorganizational, multidisciplinary group to develop an intervention to improve discharge from acute mental health services. The model of working, made up of the highest ranked ideas for improving interagency operations and communication at discharge, has potential to be piloted as an intervention to improve patient safety and experience.

## Data Availability Statement

All datasets generated for this study are included in the article/**Supplementary Material**.

## Ethics Statement

The ethics approval details are: Nottingham University Business School Research Ethics Committee (NUBS REC): Patient Safety in Mental Health Care Transitions (201819003).

## Author Contributions

NT prepared the manuscript, organized the events, collected and analyzed the data. JW and NW oversaw the project providing expert guidance within their fields, contributed to the manuscript and data collection. JW was the lead for this project. AG provided guidance throughout the project as an expert by lived experience, presented a service user perspective presentation at event 1, and drafted the manuscript. KG helped with participant recruitment, organizing the event and drafting the manuscript. SC provided guidance on manuscript and contributed to manuscript drafting and initial project planning.

## Funding

This work was funded by the National Institute for Health Research (NIHR) Greater Manchester Patient Safety Translational Research Centre (NIHR Greater Manchester PSTRC). The views expressed are those of the author(s) and not necessarily those of the NIHR or the Department of Health and Social Care.

## Conflict of Interest

The authors declare that the research was conducted in the absence of any commercial or financial relationships that could be construed as a potential conflict of interest.
