# Supplementary material for: Codesigning a Mental Health Discharge and Transitions of Care Intervention: A Modified Nominal Group Technique
Source: Front Psychiatry. 2020 Apr 21;11:328. doi: 10.3389/fpsyt.2020.00328 (PMC7186904; doi:10.3389/fpsyt.2020.00328)
Supplement: Supplementary file 1 [file DataSheet_1.docx]

**Supplementary File 1:**

**Protocol for NGT Event 1 Attendees**

This shows the numbers of staff that we aimed for to attend the event. This protocol was created considering organisational/financial pressures that prevented some professional groups from attending in large numbers (i.e. Psychiatrists/General Practitioners). When a participant dropped out we aimed to replace them with a similar professional.

| **Ward Based Staff (n=20)** | **Non-ward staff and others (n=20)** |
| --- | --- |
| Psychiatrist (n=1) | Ambulance service (n=2) |
| Occupational Therapist (n=2) | Police (n=2) |
| Administrator (n=3) | Lived Experience Expert (n=1) |
| Service Manager (n=4) | Crisis (n=4) |
| Nurse (n=8) | Liaison (n=4) |
| Healthcare Assistant (n=2) | Housing (n=3) |
|  | Primary Care Nurse (n=2) |
|  | General Practitioner (n=1) |
|  | Community Nurses (n=5) |

**Protocol for Event 2 Attendees**

| **Ward Based Staff (n=5)** | **Non-ward staff and others (n=5)** |
| --- | --- |
| Head of Nursing (n=1) | Lived experience expert (n=1) |
| Nurse/Lead nurse (n=2) | Social worker (n=1) |
| Psychiatrist (n=1) | Police officer (n=1) |
| Service Manager (n=1) | Primary care nurse (n=1) |
|  | Crisis services nurse (n=1) |
