# Supplementary material for: Codesigning a Mental Health Discharge and Transitions of Care Intervention: A Modified Nominal Group Technique
Source: Front Psychiatry. 2020 Apr 21;11:328. doi: 10.3389/fpsyt.2020.00328 (PMC7186904; doi:10.3389/fpsyt.2020.00328)
Supplement: Supplementary file 2 [file DataSheet_2.docx]

Supplementary File 2: Table to show how ideas were reduced from 23 to 10 and rationale for decisions

| Idea | Reasons for Exclusion or Inclusion |
| --- | --- |
| Idea 1: Little Red Book | - Evidence of failure of patient held records in past mental health research - Not secure, safeguarding etc. |
| Idea 2: Crisis and Respite Admissions | Include in final 10 |
| Idea 3: Nurse-led Discharges | Include in final 10 |
| Idea 4: Discharge Teams | Include in final 10 |
| Idea 5: Patient Writes Discharge Plan | Include in final 10 |
| Idea 6: Mental Health Co-ordinator in each GP practice | - Not relevant to acute discharge period specifically |
| Idea 7: Building Professional Relationships | Include in final 10 |
| Idea 8: Starting Discharge Planning from Admission | PRINCIPLE: This is an over-riding principle as opposed to an intervention |
| Idea 9: Multi-Agency Risk Management Plan | Include in final 10 |
| Idea 10: Risk sharing between housing and hospital services | Include in final 10 |
| Idea 11: Multi-agency Meetings | Include in final 10 (combined with 19 into more inclusive multi-agency meetings using technology) |
| Idea 12: Patient Contracts | - Doesn’t sit well with many participants - Coercion and patient blame |
| Idea 13: Management Practice Weeks | - Converted into idea 24- interagency buddying and shadowing meetings - Removing the managerial element participants were keen to see the pressures other teams face |
| Idea 14: Personality Disorder or Cluster 7 and 8 Pathway | - Removed as the scope is too large and it is being done elsewhere on a nationwide level |
| Idea 15: Stepdown Service from Community Mental Health | - Removed as the scope is too large and it is being done elsewhere on a nationwide level |
| Idea 16: Purposeful Admission | PRINCIPLE: this is an overarching principle of effective transitions, rather than an intervention |
| Idea 17: Admission Avoidance Care Plan | - Agreed that admission might not need to be avoided and sometimes it’s a good thing |
| Idea 18: Zero Tolerance Re-definition | Not relevant to acute discharge period specifically |
| Idea 19: Redefining MDT Meetings | - Combined with 11 |
| Idea 20: Community Services Discharge Co-ordinator | - Combined with idea 4, discharge co-ordinating team can span whole care pathway |
| Idea 21: Personal Life Coach | - Too large scope - Too vague - Not specifically relevant to discharge |
| Idea 22: Recovery College | - Removed as the scope is too large and it is being done elsewhere on a nationwide level |
| Idea 23: Self-referral to the Crisis Team | Removed as the scope is too large and it is being done elsewhere on a nationwide level |
| Idea 24: Better Understanding of other agencies through buddying and shadowing | Include in final 10 |
